# Correlation of Prostatic Artery Blood Flow Assessed by Doppler Ultrasonography with Semen Characteristics in Beagle Dogs

**DOI:** 10.3390/ani10112077

**Published:** 2020-11-09

**Authors:** Victoria Luño, Marina Servián, Felisa Martínez, María Borobia, Noelia González, Lydia Gil

**Affiliations:** 1 Department of Animal Pathology, Universidad de Zaragoza, 50013 Zaragoza, Spain; felimtz@unizar.es (F.M.); noegorti@unizar.es (N.G.); lydiagil@unizar.es (L.G.); 2 Instituto Universitario de Investigación Mixto Agroalimentario de Aragón (IA2), Universidad de Zaragoza, 50013 Zaragoza, Spain; 3 Clinical Veterinary Hospital, Veterinary Faculty, Universidad de Zaragoza, 50013 Zaragoza, Spain; servianmarina@gmail.com (M.S.); mbor@unizar.es (M.B.)

**Keywords:** prostate, vascularization, pulse Doppler, semen quality, dog

## Abstract

**Simple Summary:**

This study examined the blood flow parameters of the prostate artery before dog ejaculation assessed by pulsed-wave Doppler ultrasound (PwD) changes. The main objective of this study was to characterize the vascular parameters of the prostatic artery in beagle dogs and to explore the potential use of Doppler ultrasonography as a complementary method in dog stud selection. These findings report differences between blood flow parameters in cranial and caudal locations of the prostatic artery in comparison to subcapsular and parenchymal locations. There was a high positive correlation of peak systolic velocity with total volume and sperm concentration. In conclusion, estimation of the PwD parameters of the cranial prostatic artery may be useful in the breeding evaluation of dogs.

**Abstract:**

Pulsed-wave Doppler ultrasonography (PwD) is a method used to rapidly and noninvasively assess blood flow dynamics of the canine prostate. Modifications in gland vascularization can affect seminal plasma production and consequently sperm quality. The aim of this study was to determine the normal blood flow parameters of the prostate artery in beagle dogs and to analyze the correlations between vascular flow and semen quality characteristics. PwD was performed on five beagle dogs (5–6 years) measuring vascular features in four different locations of the prostatic artery (cranial, subcapsular, parenchymal and caudal); the measured features were peak systolic velocity (PSV), end-diastolic velocity (EDV), resistive index (RI) and pulsatility index (PI). Ejaculates were obtained using digital manipulation and semen quality was evaluated by determining macroscopic (total volume, sperm-rich fraction volume, color and pH) and microscopic (sperm motility, morphology, viability and acrosome integrity) characteristics. The values of PSV, PI and RI in cranial and caudal prostatic arteries were significantly higher than in subcapsular and parenchymal arteries (*p* < 0.05). Moreover, a positive correlation of PSV value in the cranial region of the prostatic artery with total ejaculate volume (*p* < 0.01, r = 0.612) and sperm concentration (*p* < 0.01, r = 0.587) was determined. PI index was negatively correlated with sperm concentration (*p* < 0.01, r = −0.709). In conclusion, the results suggest that the prostatic artery blood flow parameters can affect macroscopic semen quality characteristics in healthy dogs.

## 1. Introduction

Ultrasound is an imaging diagnostic tool used by many veterinarians in daily small-animal practice, including in reproductive clinics [1,2]. Conventional B-mode ultrasound allows real-time images of the internal organ structure to be obtained in a noninvasive manner and Doppler ultrasound measures the amount of blood flow through arteries and veins.

Stud-dog selection includes an ultrasonographic evaluation of the reproductive tract, based on the testicles and prostate. The prostate is the only accessory gland in the dog and its main function is to produce seminal plasma; thus, the composition of the ejaculate is modified depending on its activity [3]. Seminal plasma is composed of potassium and sodium chlorides, nitrogenous substances, citric acid, fructose, ascorbic acid, inositol, phosphates, lactic acid and components of choline [4,5,6]. These substances are an energy source of spermatozoa, protect cell integrity by pH and osmotic pressure control and avoid DNA damage [7]. Seminal plasma compounds are important for sperm viability, plasma-membrane integrity and sperm motility preservation; therefore, modifications can produce changes in seminal quality and ejaculate longevity [8].

Variations in the parenchyma and vascularization of the testicles have been analyzed by different authors to determine both pathological disorders and the influence of such variations on seminal quality [9,10,11,12]. However, the prostate is generally only examined to assess for prostatic diseases related to changes in shape, position, symmetry or echogenicity [13]. Monitoring of local blood flow by pulsed-wave Doppler ultrasonography (PwD) is a useful tool to identify physio pathological prostate conditions. Several authors have described the normal prostate dynamic flow parameters in this species, obtaining values for dogs of different breeds and ages [13,14,15]. Some factors, such as pathological disorders, age and breed, affect blood flow parameters [15,16]. In addition, vascularization patterns in the prostate artery have been determined to differ between pre- and post-ejaculation assessments [17].

Changes in the size, shape and vascularization of different prostate arteries can affect the production of seminal plasma and, consequently, the functionality and fertility of the ejaculate [14]. The aim of this study was to characterize the normal vascularization of the prostate in beagle dogs by measuring the peak systolic velocity (PSV), end-diastolic velocity (EDV), resistive index (RI) and pulsatility index (PI). In addition, we evaluated correlations between seminal quality characteristics and blood flow supply of the prostate in this dog breed.

## 2. Materials and Methods 

### 2.1. Reagents and Media

All chemicals were obtained from Sigma-Aldrich Quimica, S.A. (Madrid, Spain) unless otherwise indicated. The basic extender used to dilute canine sperm was Tris-citrate-fructose (TCF) extender containing 259 mM Trizma base, 80 mM citric acid and 69 mM fructose at 6.8 pH and an osmolality of 300/330 mOsm/Kg.

### 2.2. Animals 

The study was performed in accordance with animal welfare committee ethical guidelines and all procedures were carried out according to the Spanish Policy for Animal Protection (RD53/2013; ethical approval by University of Zaragoza n°PD04/18NE). Five fertile beagle dogs aged between 5 and 6 years were included in this study. The animals were individually housed, regularly vaccinated and fed commercial dry food with free access to water. All dogs were subjected to a physical and genital-tract (scrotum, testes, epididymis and prostate) examination, including semen collection and evaluation, 4–5 days before the beginning of the study.

### 2.3. Ultrasonographic Study

The ultrasonographic exams were performed by a single examiner with a 5–14 MHz linear transducer (Zonare, Zone Sonography^®^ Technology, Madrid, Spain) for brightness mode (B-mode) and for Doppler ultrasound scanning. Dogs were positioned in lateral recumbency without sedation and the transducer was positioned on the caudal abdomen to locate the bladder and subsequently to identify the prostate. Longitudinal and transverse B-mode views were obtained to analyze the echogenicity, contours and shape of the accessory gland.

Detection of the prostatic arteries was done using color Doppler ultrasonography and the blood flow parameters were determined by PwD before each semen collection of each dog (25 exams) [14]. The prostatic artery was measured in four different locations: cranial (vessel cranial to the gland), subcapsular (involving the capsule), parenchymal (parenchymal vessels) and caudal (vessel caudal to the gland) [15]. Once the vessels were identified, PwD was initiated and spectral tracing was collected using at least three sequential similar spectral waveforms. The blood flow parameters calculated were the peak systolic velocity (PSV), the end-diastolic velocity (EDV), the resistive index (RI) (PSV–EDV/PSV) and the pulsatility index (PI) (PSV–EDV)/mean velocity). The values obtained for the three waveforms were averaged to obtain a single mean value for each measure. All measurements were obtained with an angle of insonation < 60° [13].

### 2.4. Semen Collection and Evaluation

The three fractions of the ejaculates (sperm-poor fraction, sperm-rich fraction and prostatic fraction) were collected by digital manipulation in separate prewarmed calibrated conical tubes. A total of twenty-five ejaculates (five per dog) were obtained once a week. The macroscopic assessment of canine semen quality included the evaluation of the total volume of ejaculates (sperm-rich fraction+ prostatic fraction), sperm-rich fraction volume, color and pH (pHmeter: Basic 20 Crison, Barcelona, Spain). In addition, sperm concentration was determined using photometer (SpermaCue Minitub GmbH, Tiefenbach, Germany).

### 2.5. Microscopic Sperm Evaluation

#### 2.5.1. Computer-Assisted Sperm Motility Analysis

The motion parameters were determined using a computer-assisted sperm analysis (CASA) system (ISAS^®^; PROISER; Valencia; Spain). The samples were diluted in TCF extender at a concentration of 20 × 10^6^ sperm/mL. The parameters evaluated were total motile spermatozoa (TM%), motile progressive spermatozoa (PM%), curvilinear velocity (VCL, µm/s), straight-line velocity (VSL, µm/s), average path velocity (VAP, µm/s), linearity of the curvilinear trajectory (LIN; ratio of VSL/VCL, %) and amplitude of lateral head (ALH, µm). A 5 µL aliquot of each sperm sample was placed in a prewarmed Makler counting chamber. The setting parameters were 25 frames/s, in which spermatozoa had to be present in at least 15 frames to be counted. The sperm motility variable used in the statistical analysis was the overall percentage of motile spermatozoa (VCL > 20 µm/s). Three microscopic fields were analyzed in each sample using a phase-contrast microscope (Olympus^®^ BH12, Olympus Optical Co., Barcelona, Spain).

#### 2.5.2. Sperm Morphology

Sperm morphology was examined via light-microscopic evaluation (Olympus^®^ BH12, Olympus Optical Co., Barcelona, Spain) of smears stained with Diff-Quick^®^ (MICROPTIC S.L., Barcelona, Spain) staining. At least 200 spermatozoa per slide were counted to determine the percentage of spermatozoa with abnormal morphology.

#### 2.5.3. Sperm Plasma-Membrane Integrity 

The sperm viability was evaluated using the LIVE/DEAD^®^ sperm viability kit (Thermo Fisher Scientific, Hennigsdorf, Germany). Spermatozoa was mixed with SYBR-14 solution (10 μL/mL) and incubated at 37 °C for 10 min. the samples were then mixed with propidium iodide (PI) solution (2.4 mM) and incubated at 37 °C for 10 min. The proportion of live/dead sperm cells (200 sperm per sample) was measured at 400× magnification using a fluorescence microscope (Leica^®^ DM2500 LED, Barcelona, Spain). 

#### 2.5.4. Acrosome Status 

The acrosomal membrane integrity was assessed by fluorescein isothiocyanate conjugated with peanut agglutinin (FITC-PNA) and PI staining. Spermatozoa were mixed with FITC-PNA solution (200 μg/mL) and PI solution (500 μg/mL), kept at 38 °C for 5 min and finally fixed in paraformaldehyde (4% (*v*/*v*)) in saline solution. At least 200 spermatozoa were examined under a fluorescence phase-contrast microscope. The data corresponding to viable spermatozoa (intact plasma and acrosomal membranes; PNA−/PI−) were determined using a fluorescence microscope.

### 2.6. Statistical Analysis

The statistical analysis was performed using SPSS version 22.0 for Windows (Chicago, IL, USA). Shapiro Wilk test was used to verify the normality of the values. Data were analyzed using the non-parametric Kruskal-Wallis test. The correlations between blood flow parameters and seminal quality characteristics were determined using the Spearman correlation test. The data were expressed as mean value ± standard deviation (SD). Differences were considered statistically significant at *p* < 0.05.

## 3. Results

All dogs showed no clinical, ultrasonographic or seminal abnormalities, therefore they were considered healthy. The macroscopic and microscopic seminal characteristics are summarized in Table 1. No significant differences in any of the parameters studied in each trial were observed during the experimental study.

PwD parameters of the prostatic artery are reported in Table 2. The waveforms in the cranial and caudal locations of the prostatic artery showed a continuous flow with biphasic pattern typical of high-resistance vessels. However, in subcapsular and parenchymal locations, a monophasic blood flow pattern was found. The values of PSV, PI and RI in cranial and caudal prostatic arteries were significantly higher than those in subcapsular and parenchymal arteries (*p* < 0.05). No differences in the EDV parameter were observed between the four locations.

The values of the significant correlation coefficients between Doppler velocimetry of prostatic arteries and seminal parameters are summarized in Table 3. A positive correlation in the cranial location of the prostatic artery was found between PSV and total volume (*p* < 0.01, r = 0.612) and sperm concentration (*p* < 0.01, r = 0.587). In addition, PI index was negatively correlated with sperm concentration in the same location (*p* < 0.01, r = −0.709). There were no other significant correlations.

## 4. Discussion

In the present study, we characterized the blood flow parameters in four locations of the prostatic artery in beagle dogs. In addition, positive correlations were found between vascular flow and the prostate and macroscopic seminal characteristics of ejaculate.

Prostate examination by Doppler ultrasonography is applied to assess prostatic physio pathological conditions in human medicine. However, few publications exist describing prostate vascularization in dogs. Our results showed that PSV, PI and RI were significantly greater in the cranial and caudal prostate arteries compared to subcapsular and intraprostatic arteries, consistent with the findings of References [14,15]. This description is characteristic of parenchymal organs’ perfusion: low peripheral resistance and monophasic waves at the subcapsular and parenchymal locations and medium- to high-resistance flow with biphasic waves in the cranial and caudal locations of the artery [18]. The gradual speed reduction from the cranial to parenchymal part of the artery is due to a decrease of the vascular diameter, the formation of a network of branches and vessel penetration into the prostatic capsule all factors which affect PI and RI indices [19].

The values obtained for the different blood flow parameters were similar to those described by References [14,15] for French Bulldogs and treeing Walker Coonhounds. Nevertheless, Reference [13] obtained different vascularization data in the normal prostate across several breeds of dog. The prostate size varies depending on age, breed, body weight and sexual maturity; therefore, gland vascularization could also be affected by these variables [15,20]. In addition, prostate disorders such as benign prostatic hyperplasia, neoplasms or prostatitis increase PSV, EDV and PI indices in comparison to normal prostate [14,16]. Knowledge of the mean values of the vascularization parameters of the prostatic artery provides a useful complementary diagnostic method for different prostatic pathological conditions. It is necessary to determine the reference values of vascularization parameters in dog breeds of a similar size. In our study, we measured the values of blood flow parameters in the prostatic artery in beagle dogs, which are utilized as a standard in animal experimentation procedures.

Testicular blood flow has been studied for predicting testicular function and spermatogenesis in dogs, with contradictory results. It has been shown that RI and PI could be potential markers of sperm quality and that testicular artery blood flow velocity can positively affect the speed of spermatozoa movement [10,12]. However, Reference [21] determined that no PwD parameters were predictive of future total sperm output or percentage of live normal sperm. In infertile dogs, PSV and EDV values of testicular artery were lower in comparison to fertile ones, although RI and PI indices were not affected by fertility [11].

Recently, Reference [17] demonstrated modifications in blood flow to the prostate after ejaculation, so it is possible that vascularization influences plasma seminal production and subsequently seminal characteristics. To the best of the authors’ knowledge, no previous studies that have addressed the relationship between seminal characteristics and blood flow supply of the prostate. Our data showed a positive correlation between PSV and total ejaculate volume in the cranial location of the prostatic artery. Greater perfusion of the prostatic gland enhances prostate function, seminal plasma production, and, finally, the volume of the ejaculate. In addition, sperm concentration was positively correlated with PSV in the cranial region. Each ductus deferens has an artery (artery of vas deferens), usually derived from the prostatic artery in dogs [22]. We hypothesized that a higher blood flow supply in the prostatic artery increases ductus deference secretion and muscle contraction and a greater volume of stored spermatozoa is consequently removed during ejaculation. However, PI is inversely related to the blood flow perfusion [11] and negative correlation we found with sperm concentration in the cranial region. These blood flow parameter values should be taken into account when making comparisons with reference data because prostatic pathologies increase vascularization parameters [16,20].

## 5. Conclusions

In conclusion, blood flow parameters of PSV, PI and RI in cranial and caudal prostatic arteries were significantly higher than in subcapsular and parenchymal arteries in beagle dog. A positive correlation of PSV value in the cranial region of the prostatic artery with total ejaculate volume and sperm concentration was determined. Moreover, PI index was negatively correlated with sperm concentration. The results of this study suggest that estimation of the PwD parameters of the cranial prostatic artery could be used in breeding evaluation of dogs. However, further studies are needed to evaluate the correlations of prostatic Doppler vascular features with semen quality characteristics in other species.

## Figures and Tables

**Table 1:** Values of seminal characteristics from fresh dog semen samples (*n* = 25). Data are expressed as mean ± S.D.

Semen Quality Parameters	Mean	SD
Total volume (mL)	10.09	2.12
Prostatic fraction (mL)	2.64	0.62
Concentration (spz/mL)	247.71 × 10^6^	42.91
Total motility (%)	92.66	3.02
Progressive motility (%)	54.61	5.74
VCL (µm/s)	118.58	10.64
VSL (µm/s)	71.43	9.36
VAP (µm/s)	94.80	6.76
LIN (%)	63.25	4.83
ALH (µm)	3.42	0.26
Morphology (%)	89.41	1.33
Viability (%)	85.75	7.10
Acrosome integrity (%)	93.98	4.45

VCL: Curvilinear velocity, VSL: Straight velocity, VAP: Average velocity. LIN: Linearity; ALH: lateral head displacement. SD: Standard deviation.

**Table 2:** Values of Doppler parameters of four regions of prostatic artery (*n* = 25).

Vessel	Doppler Parameters			
	PSV (cm/s)	EDV (cm/s)	RI	PI
Cranial	20.28 ± 4.60 ^a^	4.09 ± 1.74	0.79 ± 0.11 ^a^	5.49 ± 2.97 ^ab^
Subcapsular	12.57 ± 2.84 ^b^	5.84 ± 1.26	0.61 ± 0.07 ^b^	3.53 ± 1.59 ^b^
Parenchymal	10.94 ± 3.12 ^b^	4.03 ± 1.46	0.62 ± 0.15 ^b^	2.96 ± 1.82 ^b^
Caudal	17.71 ± 4.34 ^a^	4.21 ± 1.60	0.72 ± 0.14 ^a^	4.44 ± 1.20 ^ab^

PSV: Peak Systolic Velocity; EDV: End Diastolic Velocity; RI: Resistive Index; PI: Pulsatility Index. Results are expressed as mean ± S.D. ^a,b^ Different letter indicate significant differences (*p* < 0.05).

**Table 3:** Significant correlation coefficients among seminal characteristics and blood flow parameters in cranial prostatic location (*n* = 25).

Doppler Variables	Semen Quality Parameters							
	Total Volume	Prostatic Fraction	Concentration	Total Motility	Progressive Motility	Morphology	Viability	Acrosome Integrity
PSV (cm/s)	0.612 *	0.443	0.587 *	−0.210	−0.343	−0.211	0.178	0.107
EDV (cm/s)	0.011	−0.002	−0.287	−0.154	0.120	−0.045	−0.076	−0.264
RI	0.302	0.274	−0.397	0.074	0.332	−0.046	0.149	0.029
PI	0.220	0.317	−0.709 *	−0.074	−0.432	−0.035	0.334	0.021

PSV: Peak Systolic Velocity; EDV: End Diastolic Velocity; RI: Resistive Index; PI: Pulsatility Index. * *p* < 0.01. The caudal, subcapsular and parenchymal regions showed no correlation, as well as the CASA system parameters that are not mentioned in the table.
